# Case Report: Pulmonary mucormycosis caused by *Rhizopus microsporus* in a patient with chronic obstructive pulmonary disease

**DOI:** 10.3389/fmed.2025.1544621

**Published:** 2025-04-09

**Authors:** Shanshan Wang, Yanqing Liu, Liqing Hu, Guoqing Qian, Yijun Mo

**Affiliations:** ^1^Department of Laboratory Medicine, The First Affiliated Hospital of Ningbo University, Ningbo, China; ^2^Department of Infectious Diseases, The First Affiliated Hospital of Ningbo University, Ningbo, China

**Keywords:** *Rhizopus microsporus*, pulmonary mucormycosis, chronic obstructive pulmonary disease, posaconazole, infection

## Abstract

**Background:**

We report a rare case of pulmonary mucormycosis caused by *Rhizopus microsporus*, which is rare in patients with chronic obstructive pulmonary disease. *Rhizopus microsporus* had been reported as the most common etiological agent associated with human infections, except *Rhizopus oryzae* in some studies.

**Case presentation:**

We described a case of 81-year-old man with pulmonary mucormycosis caused by *Rhizopus microsporus* but no other apparent risk factors including diabetes. The diagnosis mainly relied on sputum cultures and clinical manifestations. Despite antifungal therapy, his condition worsened, resulting in mortality.

**Conclusion:**

In this case, the patient had no underlying diseases such as diabetes or solid tumors. Clinicians should be aware of routine pathogenic microbiological tests of pulmonary mucormycosis in patients with chronic obstructive pulmonary disease. Early and aggressive treatment can lead to improved prognosis.

## Introduction

Mucormycosis is defined as an opportunistic and life-threatening fungal infection that originates from the fungi order Mucorales. It is associated with high morbidity and mortality rates in patients with immune deficiencies. Mucormycosis is the third most common invasive fungal infection following candidiasis and aspergillosis (1). The infection site of involvement of mucormycosis correlates with specific predisposing factors rhino-orbital mucormycosis occurs in uncontrolled diabetes and the recipients of hematopoietic stem cell transplant and solid-organ transplant. Pulmonary mucormycosis (PM) is the second most common manifestation of mucormycosis after nasal (2). Moreover, pulmonary mucormycosis have a rapid fatal clinical progression that can be fatal if it is untreated (3). The mortality rate of infection was high (50–70%) in recent studies. The predominant fungus species causing pulmonary mucormycosis were *Rhizopus*, *Lichtheimia*, *Cunninghamella*, and *Mucor* (4). Here, we described a case of pulmonary mucormycosis in an elderly male patient diagnosed with chronic obstructive pulmonary disease (COPD) without diabetes mellitus (DM). Furthermore, we also present the data available so far on mucormycosis in COPD. Our case report emphasized the significance of considering fungal infections in patients with COPD.

## Case presentation

The patient was diagnosed with chronic obstructive pulmonary disease (COPD) at a local hospital 30 years ago, but he had no long-term follow-up or maintenance therapy. He remained clinically stable until 20 days before admission, when he developed paroxysmal dry cough without identifiable triggers. He was presented to the emergency department with a three-day history of recurrent hemoptysis. The patient was treated with hemostasis in a local hospital, but the symptoms had not improved significantly. CT angiography enhanced examination of the pulmonary arteries revealed lung consolidation in the right upper lobe in a local hospital.

On admission, laboratory investigations revealed an elevated level of C-reactive protein (10.5 mg/L), hemoglobin of 120 g/L, neutrophil counts of 22.6 × 10^9^/L, and platelet counts of 146 × 10^9^/L. The level of alanine aminotransferase (95 U/L) was elevated, but albumin level was 36 g/L. He was not suffering from DM, hepatitis virus infections (hepatitis A, B, C, D, and E), HIV, and syphilis. RT-PCR detection for COVID19 was negative. The physical examination revealed that his temperature was 37.3°C, with a pulse of 99 bpm, hypertensive with a blood pressure of 168/87 mmHg, and respiratory rate with oxygen saturation of 95% under supplemental oxygen therapy via nasal cannula at 2 L/min on room air.

A cardiopulmonary examination noted that thickening breathing sounds in both lungs. The remainder of the physical examination was unremarkable. His oral mucosa was positive for thrush. He was discharged with the diagnosis of lung abscess, COPD with acute lower respiratory tract infection and accompanied by hypertension.

Having presented with severe symptoms of lung infection, the patient was admitted to the infectious disease department, and was empirically treated with intravenous meropenem (1 g/quaque die 8 h). In addition, bronchoscopy was recommended to identify the causative pathogens in order to determine the etiology. However, the patient explicitly declined bronchoscopy. Sputum cultures were collected before empiric treatment. To mitigate sampling limitations, we strictly followed guidelines for expectorated sputum collection, including pre-procedural oral hygiene, deep cough technique, and immediate transportation to the microbiology laboratory.

**Figure 1:**
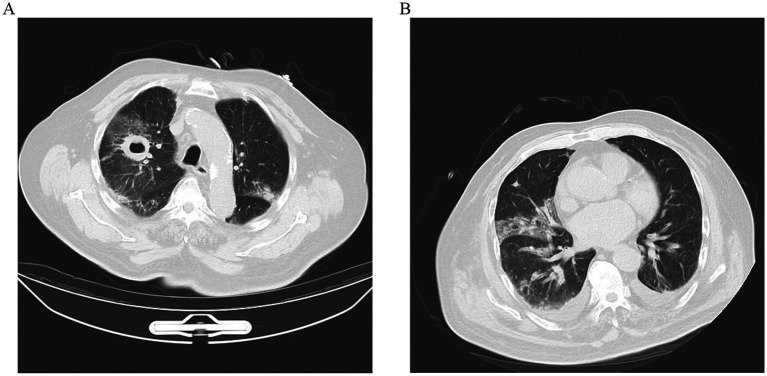
**(A)** CT scan image of upper lobes of the lungs demonstrating bilateral pulmonary infection, with cavitation (about 32 × 40 mm) in the upper lobe of the right lung, and an air sac cavity in the middle lobe of the right lung. **(B)** After 10 days of antifungal treatment, chest CT showed a decrease in lung inflammation and a diminution of cavitation (about 29 × 21 mm) in the upper lobe of the right lung.

Two days later, despite being hemodynamically stable, the patient continued to be feverish with hemoptysis. Computed tomography (CT) scan of the chest indicated that lungs appeared inflammation, including a thick-walled cavity in the upper lobe of the right lung, an air sac cavity in the middle lobe of the right lung, and arteriosclerosis (Figure 1A).

**Figure 2:**
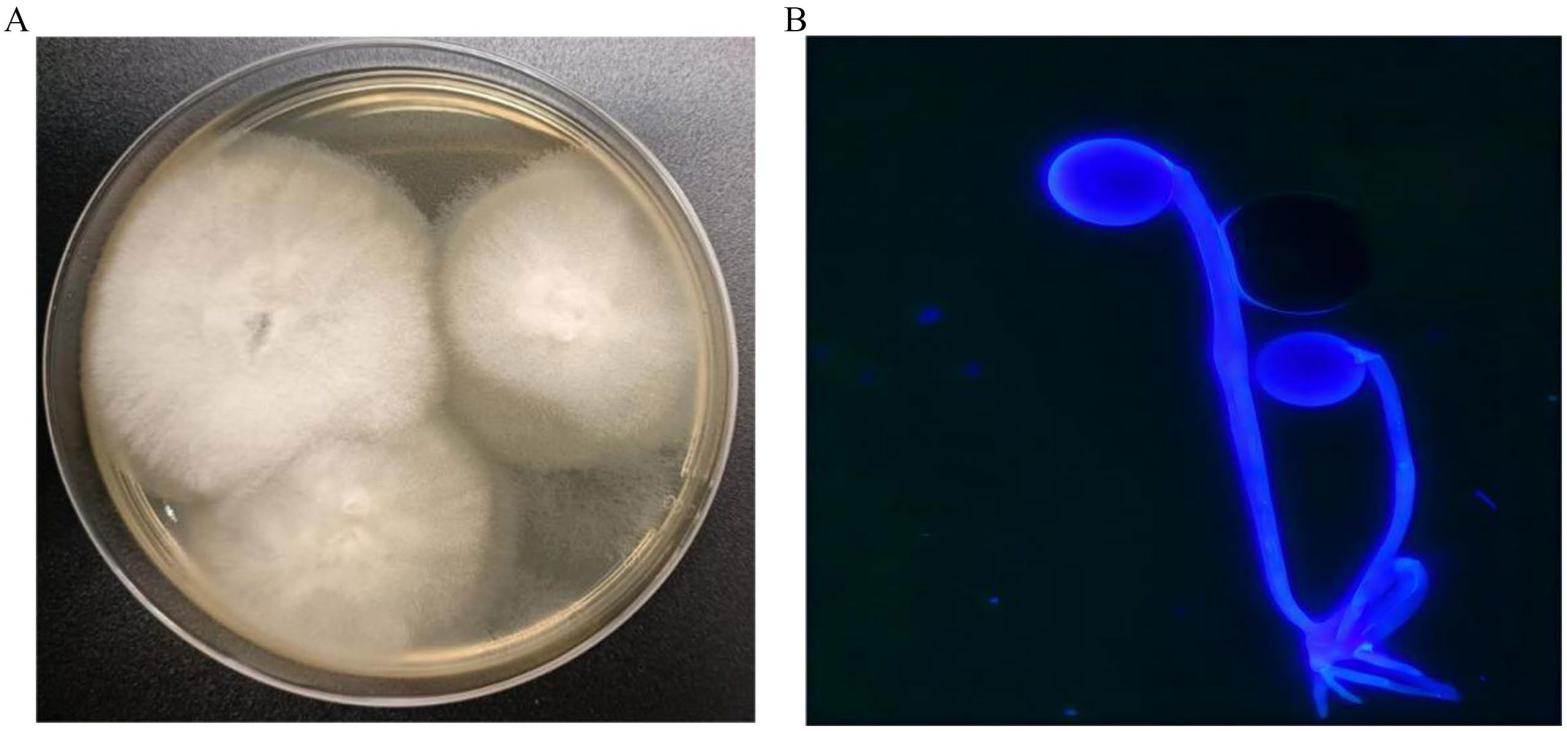
**(A)** Sabouraud dextrose agar (SDA) plate (60 mm) after 2 days incubation at 28°C, demonstrating white and woolly colonies with a short nap. **(B)** Fluorescent staining examination shows sporangiospores in pairs terminating in sporangium with subspherical columella containing brownish sporangiospores with primitive rhizoids (×400).

After 2 days of incubation, the sputum fungal culture grew white and woolly colonies with a short nap inoculated on sabouraud dextrose agar (SDA) under 28°C (Figure 2A). Fluorescent microscope examination showed shows sporangiospores in pairs terminating in sporangium with subspherical columella containing brownish sporangiospores with primitive rhizoids (Figure 2B). Three consecutive cultures of the sputum grew *Rhizopus microsporus*, which was identified by matrix-assisted laser desorption/ionisation time-of-flight mass (MALDI-TOF). For further identification, we performed the amplification of the internal transcribed spacer (ITS) gene by PCR with primer ITS1 and primer ITS4. The amplified PCR products were purified and directly sequenced through targeted DNA method. The DNA sequences were searched in GenBank by using the BLAST server. The ITS sequence demonstrated 99% identity to *R. microsporus* (GenBank accession number: No. MT 620751). Serological assay for *Aspergillus*, galactomannan, β-D-glucan, and quantitative PCR in serum sample was normal. Based on morphological characteristics (Figure 2) and sequences findings in immunocompromised states, a diagnosis of pulmonary mucormycosis (PM) was confirmed, *Rhizopus microsporus* being the most likely etiology. At the same time, antifungal susceptibility testing was performed according to the CLSI M38-A2 method (5). The results showed that the isolate was sensitive to posaconazole and amphotericin B. Meanwhile, the targeted next-generation sequencing (tNGS) of sputum was further detected. The tNGS suggests influenza A virus H3N2 (sequence number: 1674). Unexpectedly, the sequencing results did not cue that *Rhizopus microsporus*.

With the diagnosis of PM caused by *Rhizopus microsporus*, he was administered a high dosage of liposomal amphotericin B (10 mg/kg/d IV) with monitoring of renal functions and electrolytes. In addition, the patient was also treated with oseltamivir to fight the virus. However, after 5 days of antifungal therapy, the treatment was discontinued due to persistent severe hypokalemia, which was difficult to correct. Itraconazole (0.3 g/quaque die 1 day) was then initiated on day 5, accompanied by symptomatic supportive treatment for hypertension. After 10 days of antifungal therapy, repeated thoracic CT scan (Figure 1B) revealed the cavities in the lungs were significantly reduced in size, while the pleural effusion was slightly increased. But the WBC, CRP, and PCT levels remained elevated, and multiple plaques were found in the arteries of the left lower extremity. Furthermore, he remained afebrile with breathlessness. His sputum cultures showed *Acinetobacter baumannii* infection. The patient was added to intravenous meropenem (1 g/quaque die 12 h) for anti-infection treatment. Unfortunately, his symptoms of the infection did not improve and the disease progressed rapidly after 22 days of admission. He and his family decided to give up further treatment with progressive worsening of shortness of breath. The patient’s blood oxygen saturation was low at discharge, maintained at about 75–85% under oxygen inhalation. The patient died soon after discharge during the subsequent follow-up.

## Discussion

Here we present a case of pulmonary mucormycosis with COPD. Mucormycosis is a rare, rapidly progressing, and life-threatening fungal disease caused by fungi of the order Mucorales. It has commonly been seen in immunocompromised patients (1). Risk factors for mucormycosis include solid organ, stem cell transplantation, hematologic malignancies, uncontrolled DM, corticosteroids use, neutropenia, trauma, and burns. In recent years, the incidence of PM, has been increasing. Predisposing factors play an important role in mucormycosis infection. Conditions involving immunodeficiency are most prevalent risk factor, among which diabetes mellitus emerged as the most significant. Other risk factors, such as stem cell or solid organ transplant, hematological malignancy, and autoimmune diseases, cannot be ignored. Several studies have suggested an increased relative abundance of *Rhizopus microsporus* in patients with DM. Evidence showed that exposure to spores from *Rhizopus microsporus* might cause several types of respiratory symptoms in wood trimmers (6). In this case report, we emphasized the significance of considering fungal infections in patients with common diseases, including COPD. The risk factors for mucormycosis in our patient included coughing and expectorating repeatedly for more than 30 years. However, the interesting aspects of our case were the absence of DM and malignancy cause for immunosuppression. But his prognosis was still fatal. As evidenced by previous studies, *Rhizopus microsporus* is an opportunistic pathogen that could cause serious and life-threatening infections.

Diagnosis of pulmonary mucormycosis is challenging owing to its rarity. The symptoms of pulmonary mucormycosis are non-specific, such as fever, cough, dyspnea, and chest pain. Prolonged fever is mostly seen in many patients with this infection, although it might be symptomless (7). Both bronchoscopic and percutaneous lung biopsy are effective tools to help diagnose PM. But bronchoscopy was declined by the patient in this case. Previous studies have identified associations between sputum microbiology and various clinical factors in patients with COPD exacerbations (8). The microbiological reliability of sputum specimens critically depends on proper collection techniques, particularly deep-cough expectoration under supervision. As evidenced by multicentre studies, supervised deep coughing significantly increases the yield of lower respiratory tract pathogens compared to spontaneously expectorated samples (9). These results will be of value to clinicians, allowing them to select which patients with COPD exacerbation need to have sputum cultures from deep-cough expectoration. Isolation of *Rhizopus microsporus* from three sputum cultures further authenticated this diagnosis.

PM is hard to diagnose as patient presentation is similar to patients with *Aspergillus* infections. In aspergillosis, the hyphae are thinner, more organised, septate and show acute angle branching. Demonstration of broad, non-septate, ribbon-like hyphae with right-angle branching is obligatory to diagnose mucormycosis (10). Mucormycosis ECMM MSG Global Guideline Writing Group pointed out that histology remains the gold standard for the diagnosis of infection, while culture of specimens is strongly recommended for genus and species identification, and for antifungal susceptibility testing (11). So the correct identification of fungi is essential in selecting the appropriate treatment. The gold standard diagnostic technique is culture and histopathological examination is usually for complementary. The patient had hemoptysis for 3 days while he was admitted at the local hospital, which was diagnosed pulmonary aspergillosis. Hemoptysis is possible due to erosion of the pulmonary artery into the tracheobronchial tree (7). We speculated that misdiagnosis was a result of the lack of morphological discrimination between *Rhizopus microsporus*, *Rhizopus arrhizus*, and *Aspergillus*, and local laboratory conditions were limited. *Rhizopus arrhizus* and *R. microsporus* are microscopically distinguished by unbranched sporangiophores with elliptical-cylindrical spores (5–8 μm) versus branched sporangiophores bearing striated subglobose spores (3–5 μm), complemented by thermotolerance divergence (≤45°C vs. 30–35°C) (12). In the present case, given that he could not tolerate urgent surgery, definitive diagnosis was through sputum culture. Three sputum cultures during the hospitalization suggested *Rhizopus microsporus*. In recent years, NGS methods have been used to try to improve the detection and identification of pathogens and have become a topic of concern as routine pathogen identification tool (13). The identification of influenza A virus H3N2 in sputum by tNGS was achieved in the early stage. Long-term use of corticosteroids in COPD patients may lead to immunosuppression, increasing the risk of fungal infections. Inhalation of fungal spores in patients with compromised lung defenses may result in PM (14). The influenza virus can damage the respiratory epithelium, facilitating fungal invasion (15). But tNGS did not indicate *Rhizopus microsporus* infections. The reason for the failure may be that *Trichoucales* belongs to the pathogen with thicker cell wall. With low extraction rate of nucleic acid, tNGS of the sample resulted in low sensitivity and detection rate (16). Therefore, when PM was suspected clinically, it should not rely on mNGS or tNGS results simply. Isolation of fungi by culture is vital for definitive identification in the early clinical diagnosis. Clinicians should keep an eye on patients who are at high risk of acquiring this fatal disease and make early intervention strategies to reduce terrible outcomes. To achieve a more rapid diagnosis of mucormycosis, more modern laboratory tools are needed, besides improved awareness by clinicians.

PM has a rapid fatal clinical progression. Its prognosis is dependent on early diagnosis and aggressive treatment. The infection has a high mortality rate (50–70%). In recent studies, surgical resection was recommended if hemoptysis was massive (10). An unprecedented outbreak of intestinal zygomycosis due to *Rhizopus microsporus* occurred in China, and similar cases have been reported in USA and elsewhere in Europe (17). Supplementary Table S1 shows clinical characteristics and outcomes of *Rhizopus microsporus* infections in patients in recently reported clinical cases (*n* = 10), including year, country, age (y), infection type and outcome. Current guidelines recommend a combined medical and local surgical debridement to management. As antifungal agents may have poor penetration at the site of the infection and the disease is rapidly progressive and associated with a bad prognosis (11). In the medical treatment of mucormycosis, the timely initiation of a liposomal formulation of amphotericin B monotherapy is the first line treatment for mucormycosis (18). Posaconazole and isavuconazole are acceptable salvage and long-term treatment options when patients are at a high risk of amphotericin-induced nephrotoxicity (19). In this case, although the patient was immediately started on a lipid formulation of amphotericin B delivered at a higher dose through aerosolized therapy with antifungal coverage before being switched to posaconazole, our patient had an unfavorable prognosis eventually. We speculated that it might be related to the rapid course of underlying disease and angioinvasive infection by *Rhizopus microsporus*.

## Conclusion

In conclusion, this case illustrates the rapid progression of PM associated with deadly outcomes. Timely prevention, early diagnosis and symptomatic treatment are effective ways to prevent mucormycosis from spreading and to ameliorate patient outcomes despite the low survival rates in disseminated cases. Laboratory personnel and doctors should cooperate with each other and summarize clinical outcomes to improve the diagnosis and efficiency from the initial pity.

## Data Availability

The original contributions presented in the study are included in the article/Supplementary material, further inquiries can be directed to the corresponding authors.
