# Supplementary material for: Case Report: Pulmonary mucormycosis caused by Rhizopus microsporus in a patient with chronic obstructive pulmonary disease
Source: Front Med (Lausanne). 2025 Apr 9;12:1544621. doi: 10.3389/fmed.2025.1544621 (PMC12014429; doi:10.3389/fmed.2025.1544621)
Supplement: Supplementary file 1 [file Supplementary_file_1.docx]

**Table S1. Clinical characteristics and outcomes of *Rhizopus microsporus* infections in patients**

| **Year** | **Country** | **Age (y)** | **Infection Type** | **Outcome** | **Reference** |
| --- | --- | --- | --- | --- | --- |
| 2022 | China | 43 | Pulmonary mucormycos | Recovered | [1] |
| 2020 | Austria | 52 | Pulmonary mucormycos | Death | [2] |
| 2021 | France | 55 | Pulmonary mucormycos | Death | [3] |
| 2021 | China | 26 | Pulmonary mucormycos | Recovered | [4] |
| 2022 | Brazil | 67 | Rhino-orbito- cerebral mucormycosis | Recovered | [5] |
| 2017 | Thailand | 25 | Gastrointestinal mucormycosis | Death | [6] |
| 2021 | USA | 36 | Gastrointestinal mucormycosis | Recovered | [7] |
| 2022 | USA | 65 | Respiratory Tract Infection | Death | [8] |
| 2022 | China | 24 | Invasive splenic mucormycosis | Recovered | [9] |
| 2022 | China | 62 | invasive pulmonary mucormycosis | Death | [10] |

**References**

[1] X.Z. Guo, L.H. Gong, W.X. Wang, D.S. Yang, B.H. Zhang, Z.T. Zhou, and X.H. Yu, Chronic pulmonary mucormycosis caused by rhizopus microsporus mimics lung carcinoma in an immunocompetent adult: A case report. World journal of clinical cases 11 (2023) 3295-3303.

[2] C. Zurl, M. Hoenigl, E. Schulz, S. Hatzl, G. Gorkiewicz, R. Krause, P. Eller, and J. Prattes, Autopsy Proven Pulmonary Mucormycosis Due to Rhizopus microsporus in a Critically Ill COVID-19 Patient with Underlying Hematological Malignancy. Journal of fungi (Basel, Switzerland) 7 (2021).

[3] A.P. Bellanger, J.C. Navellou, Q. Lepiller, A. Brion, A.S. Brunel, L. Millon, and A. Berceanu, Mixed mold infection with Aspergillus fumigatus and Rhizopus microsporus in a severe acute respiratory syndrome Coronavirus 2 (SARS-CoV-2) patient. Infectious diseases now 51 (2021) 633-635.

[4] F. Yuan, J. Chen, F. Liu, Y.C. Dang, Q.T. Kong, and H. Sang, Successful treatment of pulmonary mucormycosis caused by Rhizopus microsporus with posaconazole. European journal of medical research 26 (2021) 131.

[5] S.D. Munhoz, R.F. Lellis, A.P.C. Reis, G.M.B. Del Negro, M.G.T. Sousa, and J.V. Veasey, Rhino-orbito-cerebral mucormycosis caused by Rhizopus microsporus var. microsporus in a diabetic patient with COVID-19. Anais brasileiros de dermatologia 97 (2022) 501-504.

[6] T. Yinadsawaphan, P. Ngamskulrungroj, W. Chalermwai, W. Dhitinanmuang, and N. Angkasekwinai, Gastrointestinal mucormycosis due to Rhizopus microsporus following Streptococcus pyogenes toxic shock syndrome in an HIV patient: a case report. BMC infectious diseases 20 (2020) 817.

[7] M. Trybula, D. Wang, L. Baumann, T.A. Pritts, and B.C. Hambley, Rhizopus microsporus typhlitis in a patient with acute myelogenous leukemia. Clinical case reports 9 (2021) e04290.

[8] S. Yang, V. Anikst, and P.C. Adamson, Endofungal Mycetohabitans rhizoxinica Bacteremia Associated with Rhizopus microsporus Respiratory Tract Infection. Emerging infectious diseases 28 (2022) 2091-2095.

[9] X. Peng, Z. Wei, L. Wang, and J. Cheng, Invasive splenic mucormycosis due to Rhizopus microsporus during chemotherapy for acute monocytic leukemia: a case report and literature review. Frontiers in oncology 13 (2023) 1237807.

[10] C. Kong, L. Zong, S. Ji, Y. Liu, and M. Li, Case report: Disseminated mucormycosis misdiagnosed as malignancy developed from allergic bronchopulmonary mycosis caused by Rhizopus microsporus following SARS-CoV-2 infection in a woman. Frontiers in medicine 11 (2024) 1394500.
